# Participatory development of a framework to actively involve people living with dementia and those from their social network, and healthcare professionals in conducting a systematic review: the DECIDE-SR protocol

**DOI:** 10.1186/s40900-023-00461-2

**Published:** 2023-07-11

**Authors:** Mike Rommerskirch-Manietta, Christina Manietta, Anna Louisa Hoffmann, Helga Rohra, Dianne Gove, Birgit Alpers, Lillian Hung, Carol R. Geary, Katherine M. Abbott, Lily Haopu Ren, Stefanie Oberfeld, Ana Diaz, Martina Roes

**Affiliations:** 1 Deutsches Zentrum für Neurodegenerative Erkrankungen (DZNE), Site Witten, Witten, Germany; 2 Department of Nursing Science, Faculty of Health, Witten/Herdecke University, Witten, Germany; 3 Deutsches Zentrum für Neurodegenerative Erkrankungen (DZNE), Patient Advisory Board, Bonn, Germany; 4 Alzheimer Europe, Luxembourg, Luxembourg; 5 Medical Center Hamburg Eppendorf (UKE), Hamburg, Germany; 6 School of Nursing, The University of British Columbia, Vancouver, Canada; 7 College of Medicine, University of Nebraska, Omaha, USA; 8 Department of Sociology and Gerontology, Miami University, Oxford, USA; 9 Scripps Gerontology Center, Oxford, USA; 10 Department of Geriatric Psychiatry, St. Rochus-Hospital, Telgte, Germany

**Keywords:** Framework, Participatory research, Collaboration, Public, Dementia

## Abstract

**Background:**

Systematic reviews summarize and evaluate relevant studies to contribute to evidence-based practice. Internationally, researchers have reached a consensus that the active involvement of the public leads to better research. Despite this agreement, there are many reviews of research concerning healthcare interventions intended to promote the care of people living with dementia and those from their social network (e.g., close contacts, both family and non-family members) primarily involve only healthcare professionals and other experts. Due to the lack of a dementia-sensitive framework to actively involve people living with dementia and those from their social network, and healthcare professionals as co-researchers in systematic reviews, it is important to develop a framework to inform practice.

**Methods:**

For this framework development process, we will recruit four people living with dementia and a total of four people from their social network, and three healthcare professionals working in acute or long-term care settings. We will conduct regular meetings with these groups of the public and healthcare professionals to include them in all stages of the systematic review. We will also identify and develop methods necessary to ensure meaningful involvement. The results will be documented and analyzed for the development of a framework. For the planning and preparation for these meetings, as well as the conduct of the meetings themselves, we will be guided by the principles of the INVOLVE approach. In addition, the ACTIVE framework will be used to guide the degree of involvement and the stage in the review process.

**Discussion:**

We assume that our transparent approach to the development of a framework to support the active involvement of people living with dementia and those from their social network, and healthcare professionals in systematic reviews will serve as an impetus for and provide guidance to other researchers with the goal of increasing researchers’ focus on this topic and facilitating systematic reviews that apply participatory approaches.

*Trial registration*: Trial registration is unnecessary as no intervention study will be conducted.

**Supplementary Information:**

The online version contains supplementary material available at 10.1186/s40900-023-00461-2.

## Abstract (plain language summary)

Systematic reviews summarize and evaluate studies on a particular topic. They provide information, for example, regarding whether an intervention is beneficial. This type of review is particularly important for healthcare professionals because they can use the results of the review to guide their actions. There is a growing awareness that the public, including people living with dementia and those from their social network (e.g., relatives, friends), need to be actively involved in the process of preparing these reviews when they are concerned with the topic of the reviews. Despite this consensus, it is often the case that only healthcare professionals are involved in such reviews. At present, no framework for the active involvement of people living with dementia and those from their social network, and healthcare professionals in systematic reviews has been developed. Therefore, we will develop such a framework together in collaboration with a range of members of the public and healthcare professionals. For this purpose, in addition to healthcare professionals, we will involve people living with dementia and those from their social network. Over the course of several meetings, we will engage in discussion with them and identify the stages of the process of conducting a systematic review in which their involvement as members of the researcher team is meaningful. We will furthermore identify the requirements associated with such an active involvement. A written report of these discussions will be produced in collaboration with the group. This will contribute towards the development of a framework for other researchers. The framework will later be made available to the public free of charge to increase awareness of this topic and to contribute towards more frequent, well-organised and meaningful involvement of people living with dementia and those from their social network, and healthcare professionals in systematic reviews.

**Supplementary Information:**

The online version contains supplementary material available at 10.1186/s40900-023-00461-2.

## Background

Systematic reviews are a key resource for healthcare professionals to deliver evidence-informed healthcare. The importance of such reviews is partly due to the fact that they assemble different studies related to a particular topic and evaluate the effectiveness of, for example, complex interventions with the aim of making recommendations either for or against their use [1–3]. On the other hand, studies focusing on implementation strategies can also be organized in terms of their implementation outcomes and reevaluated using systematic reviews, thus providing healthcare professionals with evidence-based information regarding the best possible implementation strategy for the intervention in question [4, 5].

These reviews are characterized primarily by a rigorous systematic, and transparent approach, and they impose certain requirements on the researchers who perform them to ensure appropriate quality. For example, the Cochrane Collaboration, one of the most respected organizations focused on conducting and publishing systematic reviews, notes that conducting these reviews requires at least one senior researcher who has experience conducting such reviews [6].

In addition, the call for the active involvement of the public and healthcare professionals has become increasingly “louder” in recent years [7, 8]. In terms of systematic reviews, this call often highlights the active involvement of healthcare professionals in the task of assessing the clinical relevance of identified interventions, which can be accomplished, for example, by following the Grading of Recommendations Assessment, Development and Evaluating (GRADE) approach [9]. Furthermore, the involvement of members of the public (e.g., people living with dementia) in systematic reviews often appears to be inconsistent, related only to specific individual stages of the review, and reporting about active involvement is nontransparent [10, 11]. In particular, the active involvement of people living with dementia and those from their social network often appears to be insufficient or limited only to the discussion and contextualization of the results of such reviews [10]. The present degree of noninvolvement of people living with dementia seems to be the result of certain stigmatizing prejudices [12] and is no longer based on the current state of research on this topic, as a multitude of research projects have successfully demonstrated ways in which people living with dementia can successfully become involved in such projects [13, 14]. In addition, it seems to be self-evident that the perspective of people living with dementia and those from their social network differs from other public groups in terms of their lived experiences, the challenges they face due to the symptoms related to dementia, and their need for dementia-sensitive support to facilitate their active participation in relevant studies [15]. However, their lived experience in particular is of great importance with respect to their role as active members of the research team, as this factor influences, for example, the development of research questions in a way that cannot be accomplished by any other means than the participation of people living with dementia and those from their social network [7, 16]. Previously, a few approaches to active involvement in different types of reviews have been developed; however, these approaches appear to be rather open and general as opposed to being specifically focused on (or even developed in collaboration with) people living with dementia and those from their social network [17, 18]. Consequently, the extent to which people living with dementia and those from their social network, and healthcare professionals can meaningfully be involved as co-researchers in systematic reviews and appropriate methods for their active involvement have yet to be identified.

## Aim and research questions

**Fig. 1:**
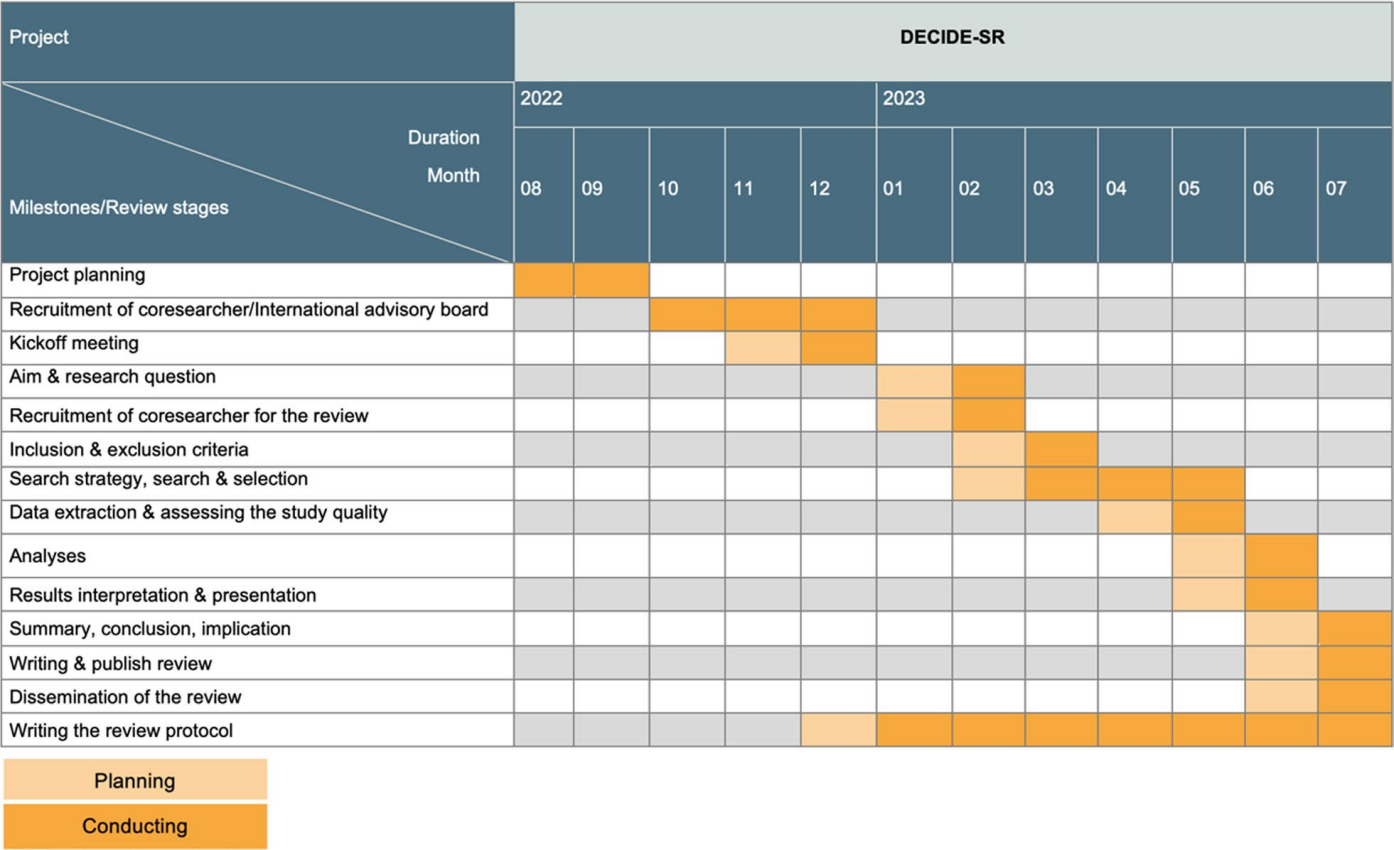
Timetable of the DECIDE-SR project

To address this research gap, we will develop a framework how to actively involve a variety of people living with dementia and those from their social network, and healthcare professionals in a systematic review in a participatory manner. This framework will have a “living” character and will be further developed through the application in future research projects based on the resulting findings and experiences. Our project will take place from August 2022 to July 2023. The planned timetable for the project is provided in Fig. 1.

We will investigate the following research questions:

### RQ1

In which stages of a systematic review can people living with dementia and those from their social network, and healthcare professionals be actively involved meaningfully, and to what extent can they be involved in the different stages?

### RQ2

Which methods are most important for people living with dementia and those from their social network, and healthcare professionals in the various stages of the systematic review to ensure their active involvement?

To report our study protocol and to ensure rigor, we will follow the short version of the GRIPP2 reporting checklists: tools to improve reporting of patient and public involvement in research [19] (Additional file 1).

## Methods

By conducting a narrative literature search for frameworks related to the involvement of public groups and healthcare professionals in research, we identified the INVOLVE briefing notes [20] and, with a specific focus on systematic reviews, the ACTIVE framework [17]. We will discuss both frameworks, adapt and use them to our task of framework development with the members of our research team (people living with dementia and those from their social network, healthcare professionals, and researcher) to support our project (DECIDE-SR).

To plan and prepare for the meetings with the public and healthcare professionals involved in our project as well as to guide the actual conduct of these meetings, we will follow the information and advice provided by the INVOLVE Briefing Notes for Researchers [20]. These notes address a total of ten different topics and aim to support and help researchers involve public groups in research. Example topics in this context include information and advice regarding planning and preparing for the process of involving the public as well as guidance for situations in which something goes wrong in the project [20]. In addition, this material contains a supplementary briefing note focused on systematic reviews [21]. This briefing note categorizes the active involvement of the public in systematic reviews into three different levels (1. in individual reviews, 2. across a group of systematic reviews, and 3. at the unit level e.g., being part of a research group/department).

The other framework that we will use and adapt for our project is the ACTIVE framework [17]. The Active framework will be used to facilitate the systematic inclusion of groups of the public and healthcare professionals in systematic reviews. For this purpose, the factors of recruitment (open or closed), approach to involvement (one-time, continuous, or combined), method of involvement (direct or indirect), the 12 stages in the review process (e.g., develop question, plan methods) and the degrees of involvement (receiving, contributing, influencing, controlling, and leading) will be taken into consideration.

### Recruitment of the research team

Since our process of framework development focuses on *individual reviews* [21], we will follow the approach of *closed recruitment* to recruit our research team [17]. This choice is particularly due to the fact that this process of framework development is embedded in the structures of the DECIDE-SR project and thus linked to a predefined time period and sources of funding. Accordingly, as early as possible in the project, a researcher (MR) will personally contact and recruit members from the Patient Advisory Board of the Deutsches Zentrum für Neurodegenerative Erkrankungen (DZNE) as well as healthcare professionals with a focus on acute care or long-term care who are collaborating with the DZNE Site in Witten. In this way, the potential people, who are interested in active involvements, will be introduced to the DECIDE-SR project and the tasks that they would face; they will also be informed that they will be financially rewarded for their *direct* and *continuous* work on the DECIDE-SR project [17, 20]. Since this project is one of the first to feature the active involvement of e.g., people living with dementia and is implemented in a situation of limited resources due to the project’s funding and time, we will ensure that the number of involved people remains small. Consequently, we determined in advance that we will recruit four people living with dementia and a total of four people from their social network, and three healthcare professionals with an expertise in dementia care representing either acute care or long-term care.

Our inclusion criteria require potential people to be members of the DZNE Patient Advisory Board or to be working in a partner facility of DZNE Site Witten. Involvement in our project DECIDE-SR does not require an assessment of the cognitive status of the people living with dementia. This decision was made because a cognitive assessment would contradict relationship building, which is an essential aspect for participatory research. Additionally, it would only refer to the actively involvement of the people living with dementia, which could cause a stigmatizing effect. Furthermore, the score of the cognitive assessment would not provide information about the skills related to the involvement in the different levels of conducting a systematic review [22].

Furthermore, potential people living with dementia who agree to work with us will receive a contract from the DZNE for their work on the DECIDE-SR project and will thus be on an equal level with the DZNE in formal terms. This contract was drafted by the DZNE legal department, clarified by the data protection department, and supervised by the compliance office. Among other things, this contract states that no personal data other than the dementia diagnosis of the research team members will be collected. The contract is discussed in detail between the people living with dementia and a DZNE researcher (MR) so that the research team members can make an informed decision. Nevertheless, all people will have the opportunity to terminate their collaboration with us at any time to ensure that they are free to decide how long and to what extent they want to be involved in the project [7].

### Planning for, preparation for and conducting of meetings

**Fig. 2:**
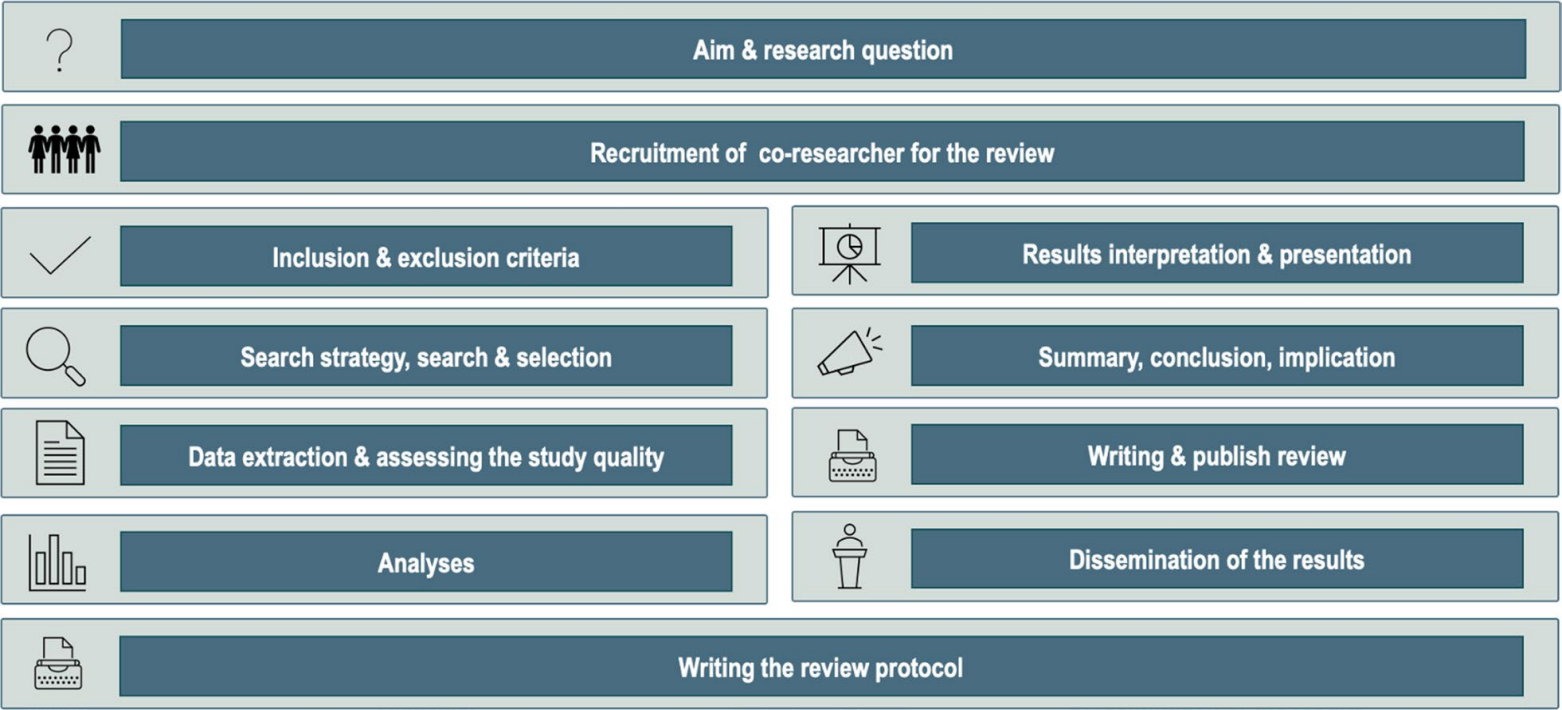
Overview of the stages to be discussed in the planned meetings

Our joint meetings, which will be structured thematically based on the stages involved in a systematic review (Fig. 2), will begin with a kickoff meeting, during which we will schedule a preparation phase and at least one meeting.

Each review stage will be discussed in at least two meetings: a decision meeting to determine the degree of involvement and one meeting to discuss the methods that are necessary to ensure the active and meaningful involvement. Preferred times and days for meetings will be requested in advance, and the times and days with the most overlap will be selected. All virtual meetings (every 14 days) will have a duration of 60–90 min, which both represents an appropriate amount of time for people living with dementia and from their social network and allows us to accommodate the time pressures frequently experienced by healthcare professionals [7, 23]. After each meeting, a protocol of the results will be written in lay terms (ALH) and emailed to the research team within one week of the meetings (MR). All information regarding an upcoming meeting (e.g., PowerPoints or relevant literature) will be prepared in lay terms and emailed to all by a researcher (MR) at least one week prior to the scheduled meeting [7].

#### Kickoff meeting

For the kickoff meeting, we will start with a personal introduction round to facilitate the establishment of a relationship within the research team as well as to reduce any initial inhibitions. As a further purpose of the kickoff meeting, two researchers (CM/MRM) will introduce the methodology associated with systematic reviews and degrees of active involvement. Subsequently, we will discuss the goal of the DECIDE-SR project and present a detailed description of the schedule for the project. Thereafter, we will discuss our expectations regarding the project, and we will establish written agreements about how we want to work together and communication rules regarding further meetings. We anticipate that not all members of the research team will be able to attend all meetings regularly and that some of them will form a core team. This possibility of dropping out situation has also been reported in the context of other public and healthcare professional groups in the context of participatory research approaches [24]. To address this issue and to “leave no one behind”, we will discuss the idea of building tandems between recruited members of the research team (people living with dementia and those from their social network, and healthcare professionals) and researchers from the DZNE at an early step of the process. Working in tandems could be implemented for each step of the DECIDE-SR study and provides the opportunity to engage in continual short exchanges on projects news between tandem partners, for example, via e-mail or telephone [25].

Additionally, we will discuss the manner in which we want to make decisions: one method could be a voting process (e.g., open versus concealed), another method could be an open consensus-based approach or a combination of different methods (such as nominal group technique) [26]. Regardless of the outcome of the discussion, there is a need to adjust the voting options (e.g., digital, or analog) individually and in consultation with the research team members, to promote an equal voting.

Finally, we plan to discuss organizational aspects of the project, such as overarching contact persons, the possibility of extra support persons, possible resources needed (e.g., internet access for virtual meetings) and channels of communication.

#### Meetings

To ensure that our meetings can determine the nature of the meaningful and active involvement in systematic reviews and the methods required to guarantee such involvement, we plan to begin each meeting with a general conversation and will ask the following questions: “Is there anything you want to share with the group before we start with our meeting?”, “Do you have any questions regarding the last or today´s meeting?”. The aim of these questions is to facilitate communication, relationship building and trust within the group and to ensure that the meeting offers a space in which everyone can talk about their current circumstances, or issues that may be bothering them now, or challenges related to the project.

**Fig. 3:**
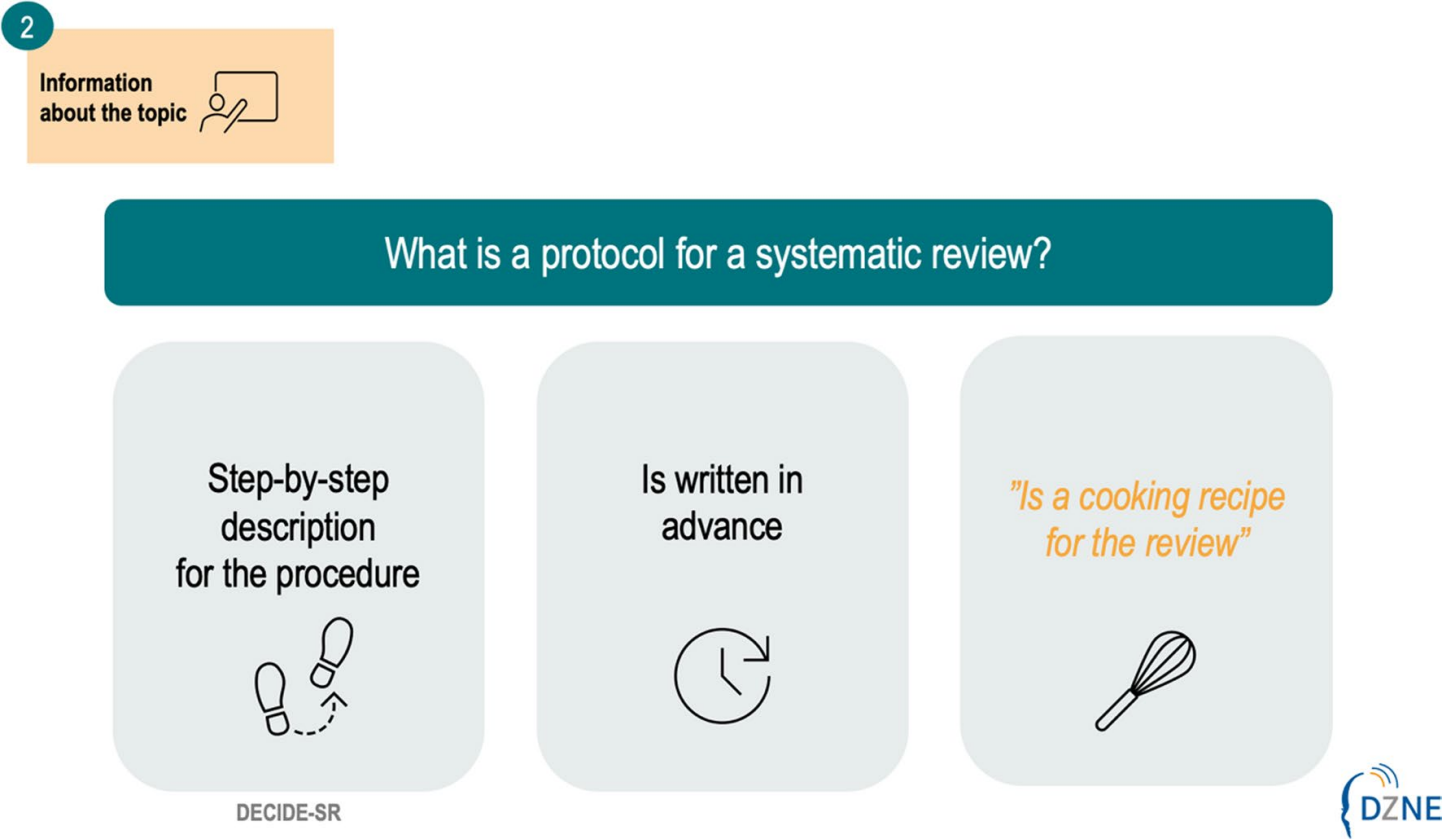
Example PowerPoint slide containing information regarding the topic at hand

With regard to the meeting focused on the task of determining the degree of active involvement, it is planned that two researchers (MRM/CM) will provide overall content information based on the corresponding stage of a systematic review (Fig. 3) as well as show and explain an example (e.g., the publication of a protocol for a systematic review). Additionally, other members of our research team can get involved in the preparation of each meeting and be involved in the presentations of content. This active involvement in the preparation and presentations will be asked at the end of each meeting for the next meeting. This offers the opportunity, that people living with dementia and those from their social network, and healthcare professionals may explain the next stage of the systematic review, e.g., development of a search string for systematic reviews.

For each degree of involvement (receiving, contributing, influencing, controlling, and leading), we will provide examples drawn from the ACTIVE framework regarding the various forms that involvement in this stage of the systematic review can take (an example can be seen in Table 1). Following the presentation of the example, the plan is for the members of the research team to vote on what they think is a meaningful degree at which they could be actively involved. The results will be discussed, and a final feedback session will be conducted to clarify any open questions or discuss further points.

**Table 1:** Examples of what involvement can look like

Degree of involvement	Example
Receiving information only	Presenting the written protocol, no active involvement besides receiving the information
Actively contributing	Discussing the written parts of the protocol, and asking for your feedback, no active involvement in the decision how to response to the provided feedback from all members of the research team
Actively influencing content	Writing together with a researcher, therefore actively involved during the writing process
Actively controlling content	Writing on your own a preferred section of the protocol and therefore defining content of the protocol
Actively leading the writing process	Acting as first or last author of the protocol

Based on the decision results for the degree of active involvement, two researchers (CM/MRM) will prepare a PowerPoint presentation for the following meeting. The focus of the meeting is on the task of discussing with the research team the methods that are appreciated to implement the decision results concerning the issue of their active and meaningful involvement. As an example, if the degree ‘influencing’ was chosen by the vote as the degree during which the review protocol will be written, we will ask them which methods they appreciate for supporting this process, such as training in the use of Microsoft Word, the publication guidelines of different journals, or the requirements of protocols based on the relevant reporting guidelines [27]. We plan to discuss these supporting methods, for example, in terms of personal attributes, and equipment. Finally, we will decide on the various support methods, and a list of the most appreciated methods will be created.

### Synthesizing, evaluating, and testing the framework

The results of these meetings will be synthesized and transformed into a framework at a final project meeting. For this purpose, the preferred degree of active involvement and supporting methods chosen by each person (as described above) will be presented in a free-of-charge, easy-to-read format (e.g., pocket cards) in a manner analogous to the corresponding stage in the process of conducting a systematic review.

Throughout the project, DECIDE-SR will be supported by stakeholders from Alzheimer Europe who are leading the European Working Group of People with Dementia (EWGPWD). Both stakeholders (DG/AD) will be consulted on a regular basis and provide critical feedback throughout the project. Their comments will be discussed with our research team. The result of this responsive feedback allows to adjust our project processes accordingly.

Finally, we expect that at the end of the framework development process, we will also have developed a protocol for conducting a systematic review written together with the research team. We want to apply this protocol in a follow-up project and will ask the people of the research team whether they are interested in continuing their active involvement of such a follow-up project.

## Discussion

Despite calls to actively involve members of the public (such as people living with dementia and those their social network) together with healthcare professionals in systematic reviews and the corresponding increase in the prevalence of this approach [28], no detailed guidance has yet been provided concerning how to achieve this goal in the context of people living with dementia and those from their social network. This research gap is surprising because the number of reviews focusing on people living with dementia is steadily increasing; nonetheless, at present, the active involvement in all research steps takes few forms other than their involvement in the discussion of review results [17].

We hope that our approach of active involvement while developing a framework to ensure active involvement of people living with dementia and those from their social network together with healthcare professionals will raise awareness among other researchers and stakeholders (e.g., funding agencies). This active involvement approach is especially important to the task of ensuring that research remains focused on the needs of people living with dementia [7, 16] and those from their social network, which may improve the provision of dementia care. Because people of the public, such as people living with dementia are expected to contribute different skills than other populations when getting actively involved in research [7], we expect that our framework will particularly offer new insights for dementia-sensitive participatory research approaches [7]. Further, this project faces a certain amount of deadline pressure, which, combined with the large number of stages that must be discussed as part of the systematic review, can lead to certain challenges. We hope to overcome these challenges by ensuring the early active involvement of all research team members and clear communication about the schedule and planned tasks. Finally, it should be mentioned that we will receive continuous support from adjacent researchers associated with the (inter)national advisory board for the project (DECIDE-SR). Based on different methodological papers about participatory research and co-research [14, 25]. We consider all members of the research team as equal co-researchers of the DECIDE-SR study. Furthermore, the DECIDE-SR study is about the development of a concept on how to actively involve people living with dementia and those from their social network, and healthcare professionals, no data will be collected and therefore no ethical clearing is necessary. Involving people living with dementia as co-researchers and not needing ethical clearing is also emphasized by Alzheimer Europe [7] as well as by the European Working Group of People with Dementia (EWGPWD) [29]. By conducting an ethical clearing due to one identity characteristic (diagnose of dementia) of one person among all co-researchers would cause discrimination and stigmatization [30]. We are aware that there might be a knowledge gap concerning ethical dilemmas while including vulnerable groups (here people living with dementia and those from their social network) as co-researchers. To be aware and act accordingly is of particular importance when people are involved based on their lived experience with dementia. Within the DECIDE-SR study we can rely on a long-term relationship and will implement a sequence of reflecting of the active involvement in each meeting [31]. Members of the DECIDE-SR research team will either be financially compensated for attending the meetings (people living with dementia and those from their social network) or attending the meetings will count as work time (healthcare professionals). The co-researchers (people living with dementia and those from their social network) will be recruited through the DZNE Patient Advisory Board and thus have been in contact with the researchers for a long time and have already collaborated in previous projects. People living with dementia will sign a contract with the DZNE, due to their potential exposure of their disease when their name will be displayed while becoming a co-author. This contract is discussed in detail with the people so that they can make an informed decision. Additionally, there is the chance to participate in the project without a contract and participating anonymously as a co-author.

### Supplementary Information

**Additional file 1.** GRIPP 2 short form.

## Data Availability

All data generated or analyzed as part of this study will be included in an upcoming article reporting the results of the study or in the Additional files associated with that article.
