# Supplementary material for: Participatory development of a framework to actively involve people living with dementia and those from their social network, and healthcare professionals in conducting a systematic review: the DECIDE-SR protocol
Source: Res Involv Engagem. 2023 Jul 11;9:52. doi: 10.1186/s40900-023-00461-2 (PMC10337195; doi:10.1186/s40900-023-00461-2)
Supplement: Supplementary file 1 — Additional file 1. GRIPP 2 short form. [file 40900_2023_461_MOESM1_ESM.pdf]

GRIPP2 short form

Section and Topic	Item	Reported on page No
1: Aim	Report the aim of PPI in the study	6
2: Methods	Provide a clear description of the methods used for PPI in the study	7-12
3: Study results	Outcomes—Report the results of PPI in the study, including both positive and negative outcomes	12
4: Discussion and conclusions	Outcomes—Comment on the extent to which PPI influenced the study overall. Describe positive and negative effects	12-14
5: Reflections/critical perspective	Comment critically on the study, reflecting on the things that went well and those that did not, so others can learn from this experience	12-14

PPI=patient and public involvement
